# Assessing the Impact of Composite Dietary Antioxidant Index on Gastric Cancer Risk: A Case–Control Study in Southeast China

**DOI:** 10.3390/nu17213473

**Published:** 2025-11-04

**Authors:** Xinyu Chen, Qingying Wang, Fengqin Zou, Yaqing Wu, Sifang Li, Wanling Zeng, Yulan Lin

**Affiliations:** Fujian Provincial Key Laboratory of Environment Factors and Cancer, Department of Epidemiology and Health Statistics, School of Public Health, Fujian Medical University, Fuzhou 350122, China

**Keywords:** gastric cancer, composite dietary antioxidant index, antioxidants, dietary nutrition, case–control study

## Abstract

**Objective**: To examine the association between the composite dietary antioxidant index (CDAI) and gastric cancer (GC) risk among adults in Southeast China, and to provide evidence for region-specific nutritional interventions. **Methods**: In this case–control study (July 2023–November 2024), 336 newly diagnosed GC patients were recruited from a hospital in Southeast China, and 336 sex-matched healthy controls were selected from local communities. Dietary data from a validated food frequency questionnaire were used to calculate CDAI scores. **Results**: A total of 672 participants (56.5% male) were included. The mean CDAI value was 0.47 ± 4.23 in cases versus −0.04 ± 4.61 in controls (*p* = 0.134), but CDAI quartile distribution differed significantly (*p* = 0.009). In multivariable analysis of individual CDAI components, vitamin C intake demonstrated a significant inverse association with GC risk, with the strongest protective effect observed in the highest quartile (OR = 0.48, 95% CI: 0.30–0.77, *p* = 0.002). Selenium intake also showed significant protective effects in the second (OR = 0.52, 95% CI: 0.32–0.83, *p* = 0.006) and third quartiles (OR = 0.50, 95% CI: 0.30–0.82, *p* = 0.006). Compared with the lowest quartile, adjusted odds ratios (95% CI) for GC in the second, third, and fourth CDAI quartiles were 0.56 (0.36–0.87), 0.59 (0.38–0.90), and 0.60 (0.39–0.92), respectively. The inverse association was stronger in participants aged >55 years, unmarried, and nonsmokers. Restricted cubic spline analysis revealed a significant nonlinear dose–response relationship. **Conclusions**: Higher dietary antioxidant intake is associated with lower GC risk. Personalized dietary strategies to enhance antioxidant intake may be particularly beneficial in high-risk populations.

## 1. Introduction

Gastric cancer (GC) is a major global public health challenge, ranking as the second leading cause of cancer-related mortality worldwide [1]. By 2050, the global burden of GC is projected to reach 2.5 million new cases and 1.9 million deaths annually [2]. China accounts for nearly 40% of the global GC burden, with 358,000 new cases and 187,000 deaths reported in 2022 [3]. In Fujian Province, the GC incidence rate in 2019 was 28.31 per 100,000 population, accounting for 9.3% of all malignancies and ranking fifth among cancer types, while the mortality rate was 20.88 per 100,000, accounting for 12.5% of all cancer deaths and ranking third [4].

Reactive oxygen species (ROS), generated under oxidative stress, are recognized as important contributors to the initiation and progression of GC [5]. Dietary factors can increase oxidative stress, leading to ROS accumulation, DNA damage, and ultimately carcinogenesis [6,7]. Unhealthy dietary patterns have been significantly associated with an elevated risk of GC [8,9], whereas foods rich in antioxidants—such as vitamin C and carotenoids—can modulate metabolic pathways, attenuate the genotoxic effects of ROS, and exert protective effects against GC [10,11,12]. Multiple studies have suggested that adequate dietary antioxidant intake may reduce GC incidence and prevalence [13,14,15]. Evidence from meta-analyses confirms that dietary intake of antioxidant vitamins, including vitamin C, vitamin E, and carotenoids, is associated with a reduced risk of gastric cancer [16,17]. However, the associations for blood levels of these antioxidants remain inconsistent, underscoring the complexity of assessing antioxidant exposure and its relationship with cancer risk. Moreover, adequate dietary antioxidant intake is not only associated with a reduced risk of specific cancers, such as gastric cancer, but also plays a crucial role in maintaining immune homeostasis and promoting overall health [18].

Given the complex etiology of GC, preventive measures can substantially reduce its mortality [19,20]. Among these, dietary factors have become an important target for primary prevention due to their clear etiological role and modifiability. However, most existing studies have focused on single nutrients or individual food components, without adequately addressing the synergistic effects of multiple dietary antioxidants.

The composite dietary antioxidant index (CDAI) is a novel measure that integrates the intake of various antioxidants—including vitamins, minerals, and phytochemicals—to provide a comprehensive assessment of overall dietary antioxidant exposure [21]. This integrated approach offers a key advantage over single-nutrient analyses by capturing the cumulative and potentially synergistic biological effects of dietary antioxidants, which may be overlooked when components are examined in isolation. In recent years, several studies have explored the association between CDAI and cancer risk. For instance, a case–control study in Iran found a significant inverse association between the dietary antioxidant index (DAI) and GC risk (OR = 0.64, 95% CI: 0.43–0.95), although the association was no longer statistically significant when analyzed in subgroups [22]. In lung cancer research, individuals in the highest quartile of a food-based CDAI had a significantly lower risk compared with those in the lowest quartile (HR_Q4 vs. Q1 = 0.64, 95% CI: 0.52–0.79) [23]. Similarly, a prospective population-based cohort study reported that participants in the highest quartile had a 20% lower risk of colorectal cancer compared with the lowest quartile (HR_Q4 vs. Q1 = 0.80, 95% CI: 0.66–0.98) [24], and higher CDAI scores were also associated with a significant reduction in cancer-related mortality (HR = 0.84, 95% CI: 0.82–0.88) [25]. An Italian study further indicated an inverse trend between CDAI and cervical intraepithelial neoplasia, though this did not reach statistical significance [26].

Although evidence suggests that CDAI may be associated with the risk of various cancers, studies specifically addressing its relationship with GC are limited, particularly in high-incidence regions such as southeastern China. Therefore, the present study aimed to evaluate the association between CDAI and GC risk in a Chinese population and to explore its potential role in primary prevention, thereby providing a scientific basis for developing region-specific nutritional intervention strategies.

## 2. Methods

### 2.1. Study Design and Study Participants

This 1:1 sex-matched case–control study was carried out in Fujian Province, China. The case group comprised 336 individuals with a new histopathological or cytological diagnosis of gastric cancer (GC), recruited from Fujian Medical University Union Hospital between July 2023 and November 2024. The control group consisted of 336 healthy residents, matched by sex and recruited from nine prefecture-level cities in Fujian during the same period. All participants (cases and controls) were required to be between 18 and 75 years of age and be local residents, defined as having lived in Fujian for a minimum of six months in the year prior to enrollment. Additionally, all participants had to be capable of effective communication and provide written informed consent. Key exclusion criteria for both groups encompassed a history of malignancy (with controls also excluding other major conditions like stroke or psychiatric disorders) and extremes of daily energy intake (females: <500 or >3600 kcal; males: <600 or >4200 kcal).

### 2.2. Questionnaire

#### 2.2.1. Food Frequency Questionnaire (FFQ)

Dietary intake information was collected using a validated structured semi-quantitative food frequency questionnaire (FFQ), the details of which have been described in our previous publication [27].

#### 2.2.2. Demographics and Lifestyles

We systematically collected comprehensive covariate data beyond dietary intake. This encompassed general demographics (age, sex, height, weight, household income, education, occupation, and perceived daily stress), from which body mass index (BMI) was derived. Additionally, we documented key lifestyle habits, specifically smoking and alcohol consumption over the preceding year. Smoking status was ascertained based on a history of either smoking ≥1 cigarette daily for over six consecutive months or a cumulative lifetime consumption of ≥150 cigarettes. Similarly, alcohol drinking was defined as consuming alcoholic beverages at least weekly for a duration exceeding six months.

### 2.3. Calculation of the Composite Dietary Antioxidant Index (CDAI)

We adapted the CDAI proposed by Wright et al. [28]. Prior to calculating the CDAI, nutrient intakes were not energy-adjusted, with the aim of ensuring direct comparability with existing CDAI literature. Specifically, this method includes seven dietary antioxidants: vitamin A, vitamin C, vitamin E, zinc, selenium, manganese, and β-carotene. Consistent with existing calculation methods, the CDAI was obtained by summing the Z-scores of the dietary intakes of these seven antioxidants. The specific calculation formula is as follows:

$$CDAI=\sum_{i=1}^{n=7}\frac{Individual\;Intake-Mean}{SD}$$

### 2.4. Statistical Analysis

Continuous variables with a normal distribution were described using the mean and standard deviation (SD), whereas those not conforming to a normal distribution were expressed as the median (M) and interquartile range (P25, P75). Categorical variables were presented as frequency and percentage (N, %). The chi-square test was used to compare categorical variables between groups, while the t-test or analysis of variance (ANOVA) was applied for continuous variables. Dietary antioxidants and CDAI were categorized into quartiles according to their distribution in the control group. Logistic regression models were employed to assess the associations between individual dietary antioxidants, CDAI, and GC risk, estimating odds ratios (ORs) with corresponding 95% confidence intervals (CIs). In addition, a linear trend test was conducted to evaluate the dose–response relationship of GC risk across CDAI quartiles. To further explore the effect of CDAI on GC risk in subpopulations with different characteristics, stratified analyses were performed. Stratification variables were selected from demographic characteristics that were statistically significant in the univariate analysis and associated with GC risk. Restricted cubic spline (RCS) regression was applied to visualize potential nonlinear relationships between CDAI and GC risk. All *p*-values were derived from two-sided tests, and a significance level of 0.05 was considered statistically significant. Data analyses were performed using the Statistical Package for the Social Sciences, version 26.0 (SPSS 26.0).

### 2.5. Ethical Considerations

This study was conducted in accordance with the principles of the Declaration of Helsinki and was approved by the Ethics Committee of Fujian Medical University (FJMU No. 2020[53]). Before participation, the purpose and content of the study were fully explained to the patients, and informed consent was obtained. Participants were free to withdraw from the study at any time if they experienced any discomfort, and refusal to participate had no impact on their medical care. All personal information of the participants was kept strictly confidential at all times.

## 3. Results

### 3.1. Baseline Demographics

Table 1 presents the baseline characteristics of the study population (N = 672). Compared with controls, GC cases were older (mean age: 56.76 ± 10.34 vs. 53.86 ± 11.13 years, *p* < 0.001) and more likely to be married (*p* = 0.011), current smokers (*p* = 0.019), and to report low or no daily life stress (*p* < 0.001). No significant differences were observed between the two groups in terms of sex distribution, education level, occupation, household income, alcohol consumption, or BMI. The mean CDAI value was slightly higher in the case group (0.47 ± 4.23) than in the control group (−0.04 ± 4.61), but the difference was not statistically significant (*p* = 0.134). In the distribution of CDAI quartiles, participants in the case group were more likely to be in the lowest quartile (Q1, <−1.95) compared with controls (36.9% vs. 25.0%), while a higher proportion of controls were in the second, third, and fourth quartiles (each 25.0%) compared with cases (19.6%, 20.8%, and 22.6%, respectively). The difference in CDAI distribution between the two groups was statistically significant (*p* = 0.009).

### 3.2. Association Between CDAI Components and Gastric Cancer Risk

Table 2 shows the associations between individual CDAI components and GC risk. In multivariable logistic regression models adjusted for vitamin A, vitamin C, vitamin E, selenium, manganese, age group, marital status, smoking, and daily life stress level, higher intakes of vitamin C and selenium were significantly associated with a reduced risk of GC. Compared with the lowest quartile, participants in the highest quartile of vitamin C intake had a 52% lower risk of GC (OR = 0.48, 95% CI: 0.30–0.77, *p* = 0.002), while those in the second and third quartiles of selenium intake had ORs of 0.52 (95% CI: 0.32–0.83, *p* = 0.006) and 0.50 (95% CI: 0.30–0.82, *p* = 0.006), respectively. No significant associations were observed for vitamin A, vitamin E, zinc, manganese, or β-carotene after adjustment.

### 3.3. Association Between CDAI Score and Gastric Cancer Risk

Table 3 summarizes the association between CDAI quartiles and GC risk. In the unadjusted model, higher CDAI scores were significantly associated with a lower risk of GC, with ORs (95% CIs) of 0.53 (0.35–0.81), 0.57 (0.37–0.86), and 0.61 (0.40–0.93) for the second, third, and fourth quartiles, respectively, compared with the lowest quartile (*p* for trend = 0.020). Similar inverse associations were observed after adjusting for age group, marital status, daily life stress level, and smoking, with adjusted ORs (95% CIs) of 0.56 (0.36–0.87), 0.59 (0.38–0.90), and 0.60 (0.39–0.92) for the second, third, and fourth quartiles, respectively (*p* for trend = 0.019).

Table 4 presents the stratified analysis of the association between CDAI scores and GC risk. In participants aged >55 years, higher CDAI scores were significantly associated with reduced GC risk, with adjusted ORs (95% CIs) of 0.48 (0.26–0.90) and 0.42 (0.23–0.76) for the second and third quartiles, respectively (*p* for trend = 0.044). Among married individuals, CDAI scores in the third quartile were associated with a borderline significant risk reduction (OR = 0.64, 95% CI: 0.41–1.00, *p* = 0.046). In the subgroup of participants with low or no daily life stress, the third quartile of CDAI was associated with a significantly lower GC risk (OR = 0.49, 95% CI: 0.28–0.85, *p* = 0.019). Similarly, among participants with moderate or high stress levels, those in the second quartile had a significantly reduced GC risk (OR = 0.37, 95% CI: 0.18–0.75, *p* < 0.050). No statistically significant associations were observed in other subgroups.

### 3.4. Dose–Response Relationship Between CDAI and Gastric Cancer Risk

**Figure 1:**
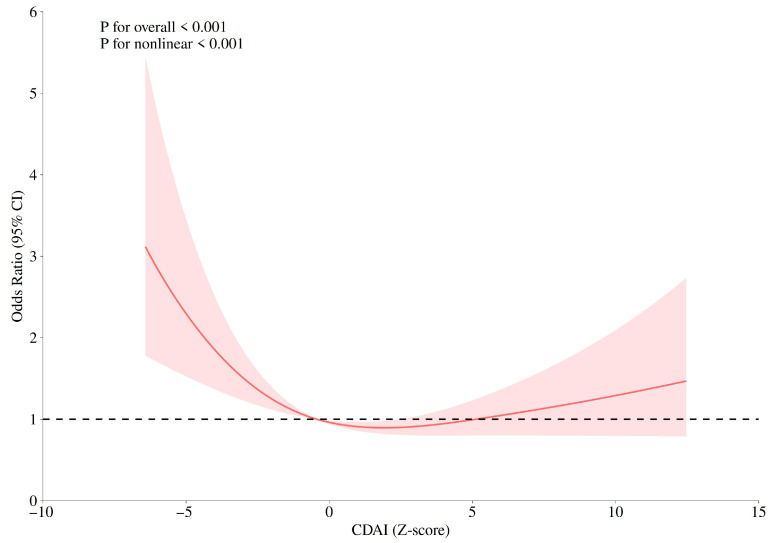
Restricted cubic spline (RCS) plots for the correlations between CDAI and gastric cancer risk.

Restricted cubic spline (RCS) models were used to evaluate the dose–response relationship between CDAI and GC risk. Knots were placed at the 10th, 50th, and 90th percentiles of the CDAI distribution, with the 50th percentile as the reference. The models were adjusted for age group, marital status, daily life stress level, and smoking. As shown in Figure 1, a significant nonlinear association was observed between CDAI and GC risk (*P*_for overall_ < 0.001, *P*_for nonlinearity_ < 0.001), with a U-shaped curve indicating that both low and high CDAI levels were associated with higher GC risk, whereas moderate CDAI values were associated with the lowest risk.

## 4. Discussion

The present case–control study aimed to investigate the association between the composite dietary antioxidant index (CDAI) and the risk of gastric cancer (GC). Our findings indicate that higher CDAI scores, reflecting diets rich in antioxidant nutrients, were significantly associated with a lower risk of GC, and this relationship remained robust after adjustment for multiple potential confounders. In addition, higher intakes of vitamin C and selenium were independently associated with reduced GC risk. Stratified analyses suggested that the inverse association between CDAI and GC risk was more pronounced among participants aged >55 years, those not currently married, and nonsmokers. Furthermore, RCS analysis revealed a significant nonlinear dose–response relationship between CDAI and GC risk, characterized by a U-shaped curve. These results highlight the potential role of dietary antioxidant status in GC prevention and underscore the importance of dietary interventions targeting oxidative stress in high-risk populations.

### 4.1. Association of CDAI Components with Gastric Cancer

GC development is influenced by genetic, infectious, environmental, and lifestyle factors, among which dietary nutrition plays a particularly important and modifiable role. In the present study, vitamin C and selenium intake were inversely associated with GC risk.

Biologically, vitamin C is a major water-soluble antioxidant that scavenges reactive oxygen species (ROS) and mitigates oxidative DNA damage in the gastric mucosa [29]. It also competitively inhibits the reaction between nitrite and amines in the stomach, thereby blocking the endogenous formation of carcinogenic *N*-nitroso compounds [30]. Our findings are consistent with previous epidemiological evidence. A meta-analysis of 32 prospective studies reported that high vitamin C intake reduced GC risk by 19% (OR = 0.81, 95% CI: 0.68–0.95), with dose–response analysis suggesting an optimal protective effect at 65 mg/day [17]. Similarly, a Korean case–control study observed significantly lower vitamin C intake among GC patients, with an inverse association between intake and GC risk (OR = 0.64, 95% CI: 0.46–0.88) [31].

Selenium intake was also significantly inversely associated with GC risk in our study, particularly in the third quartile, where the strongest protective effect was observed. Selenium’s anticancer effects are primarily mediated through its role as an essential component of selenoproteins [32]. These selenium-dependent antioxidant enzymes, such as glutathione peroxidases (GPx) and thioredoxin reductases (TrxR), protect gastric mucosal DNA from oxidative damage and modulate tumor-related signaling pathways, including NF-κB and p53 [33]. The attenuated and nonsignificant protective effect observed in the highest quartile may reflect selenium’s biphasic dose–response, whereby excessive intake could induce pro-oxidant effects [34,35]. Evidence from a dose–response meta-analysis on prostate cancer supports this phenomenon [36]. Specifically, the protective effect was observed at plasma/serum selenium concentrations up to ~170 ng/mL and toenail selenium levels between 0.85–0.94 μg/g, respectively, beyond which the benefit diminished. Furthermore, the protective association of CDAI may extend beyond essential vitamins and minerals to include non-vitamin antioxidants. Polyphenols such as resveratrol, for instance, contribute to antioxidant and anti-inflammatory defense through distinct mechanisms like Nrf2 pathway activation, and may synergize with other lifestyle factors for broader health benefits [37].

### 4.2. Association Between CDAI and Gastric Cancer

CDAI, an integrative measure of dietary antioxidant capacity, was constructed in this study from seven key antioxidant nutrients. In multivariable-adjusted models, participants in the highest CDAI quartile had a significantly lower risk of GC compared with those in the lowest quartile, suggesting that antioxidant-rich dietary patterns may protect against GC.

Mechanistically, oxidative stress contributes to carcinogenesis via DNA damage, gene mutations, and dysregulated cell signaling [38]. High CDAI scores indicate diets rich in antioxidants, which can promote apoptosis of damaged cells, suppress malignant transformation, and scavenge ROS within cancer cells [39,40]. Moreover, dietary antioxidants may exert epigenetic effects by modulating DNA methylation, histone modifications, and noncoding RNAs, thereby influencing GC-related gene expression [41,42]. These mechanistic insights provide biological plausibility for the observed inverse association between CDAI and GC risk.

Previous studies have linked higher intake of individual CDAI components to reduced GC risk [43,44,45], while cohort studies in the United States have reported lower cancer mortality among individuals consuming antioxidant-rich diets [46]. Beyond cancer prevention, higher CDAI has also been associated with reduced risk of chronic diseases and improved health outcomes [47,48].

### 4.3. Population Heterogeneity in the CDAI–Gastric Cancer Association

Our stratified analyses revealed potential population heterogeneity in the CDAI–GC relationship. The inverse association was stronger in older adults (>55 years), possibly because endogenous antioxidant defense systems decline with age, making dietary supplementation more impactful [49]. Among unmarried individuals, the protective effect was more pronounced, whereas in married participants, social support and associated health behaviors may attenuate the relative contribution of dietary antioxidants [50]. In nonsmokers, the protective association was also more evident, potentially because tobacco smoke–induced oxidative stress and nitrosamine exposure impair antioxidant response pathways, such as Nrf2 signaling [51]. Notably, the protective effect of higher CDAI was consistent across stress-level subgroups, suggesting broad applicability of antioxidant-focused dietary interventions.

RCS analysis further demonstrated a significant nonlinear dose–response between CDAI and GC risk, with a U-shaped curve indicating the lowest risk at moderate CDAI levels. It was also demonstrated that there was no significant difference in the mean CDAI scores between the case group and the control group; however, analysis based on quartiles revealed a significant negative correlation trend. This pattern suggests that while moderate antioxidant intake may effectively neutralize excessive ROS and maintain redox homeostasis, excessive intake could disrupt physiological ROS signaling or induce pro-oxidant effects [52]. These findings emphasize the importance of determining optimal antioxidant intake levels for personalized dietary prevention strategies.

### 4.4. Limitations

Several limitations should be acknowledged. First, as a case–control study, dietary data were retrospectively collected using a food frequency questionnaire (FFQ), which is subject to recall bias—particularly among GC patients, who may overreport unhealthy dietary habits. Although we instructed all participants to recall their dietary habits from the year prior to diagnosis (for cases) or interview (for controls), the accuracy of recall may still differ between groups. Second, we were unable to account for *Helicobacter pylori* infection, an important confounder in GC risk assessment. Third, due to clinical data limitations, we could not perform subgroup analyses based on GC pathological subtypes, which may have provided additional insights into differential associations. Fourth, the generalizability of our findings may be limited. The use of community-based controls and the specific dietary patterns and antioxidant sources unique to Southeast China may restrict the direct extrapolation of our results to other populations. Finally, information on participants’ physical activity levels was not collected. Since physical activity can influence oxidative stress and overall health, future studies could benefit from incorporating such data to further clarify the independent role of dietary antioxidants.

## 5. Conclusions

In summary, this case–control study demonstrated that higher CDAI scores, indicative of antioxidant-rich diets, were significantly associated with lower GC risk. Increased consumption of vitamin C- and selenium-rich foods may represent effective dietary strategies for GC prevention, particularly in high-risk populations and regions with high GC incidence. These findings provide scientific evidence for dietary prevention and intervention strategies targeting oxidative stress in GC. Public health initiatives should integrate antioxidant-rich dietary guidance into chronic disease prevention frameworks, especially in GC-endemic regions. Community-level interventions, including systematic nutritional assessments, personalized dietary recommendations, and early nutritional risk screening, may enhance public awareness of the link between dietary antioxidants and cancer risk. Future randomized controlled trials are warranted to confirm the preventive effects of optimized CDAI on gastric precancerous lesions and to explore potential interactions among its components, thereby advancing precision nutrition approaches in GC prevention.

## Figures and Tables

**Table 1:** General demographic information and distribution of CDAI scores of the study population (N = 672).

Variables	Total (N = 672)	Cases (N = 336)	Controls (N = 336)	*p* Value
Age, years, mean ± std	55.31 ± 10.83	56.76 ± 10.34	53.86 ± 11.13	<0.001
Age groups, years				
≤55	320 (47.6)	132 (39.3)	188 (56.0)	<0.001
>55	352 (52.4)	204 (60.7)	148 (44.0)	
Sex				1.000
Male	380 (56.5)	190 (56.5)	190 (56.5)	
Female	292 (43.5)	146 (43.5)	146 (43.5)	
BMI (kg/m^2^)				0.068
<24	415 (61.8)	196 (58.3)	219 (65.2)	
≥24	257 (38.2)	140 (41.7)	117 (34.8)	
Marital status				0.011
Married	611 (90.9)	315 (93.8)	296 (88.1)	
Single/Separated/Divorced/Widowed	61 (9.1)	21 (6.2)	40 (11.9)	
Education level				0.157
Primary school or below	276 (41.1)	143 (42.6)	133 (39.6)	
Secondary school	177 (26.3)	90 (26.8)	87 (25.9)	
High school	105 (15.6)	58 (17.3)	47 (14.0)	
College	51 (7.6)	20 (5.9)	31 (9.2)	
University or above	63 (9.4)	25 (7.4)	38 (11.3)	
Occupation				0.361
Farmers/Manual workers	199 (29.6)	99 (29.5)	100 (29.8)	
Other occupations	239 (35.6)	112 (33.3)	127 (37.8)	
Homemakers/Retired/Unemployed	234 (34.8)	125 (37.2)	109 (32.4)	
Average monthly household income, RMB				0.167
<3000	64 (9.5)	26 (7.7)	38 (11.3)	
3000–6000	229 (34.1)	123 (36.6)	106 (31.6)	
>6000	379 (56.4)	187 (55.7)	192 (57.1)	
Smoking				0.019
Yes	233 (34.7)	131 (39.0)	102 (30.4)	
No	439 (65.3)	205 (61.0)	234 (69.6)	
Alcohol drinking				0.916
Yes	107 (15.9)	53 (15.8)	54 (16.1)	
No	565 (84.1)	283 (84.2)	282 (83.9)	
Daily life stress				<0.001
None/Low	409 (60.9)	227 (67.6)	182 (54.2)	
Moderate/High	263 (39.1)	109 (32.4)	154 (48.8)	
CDAI, mean ± std	0.22 ± 4.43	−0.04 ± 4.61	0.47 ± 4.23	0.134
CDAI				0.009
Q1 (<−1.95)	208 (31.0)	124 (36.9)	84 (25.0)	
Q2 (−1.95 to−0.12)	150 (22.3)	66 (19.6)	84 (25.0)	
Q3 (−0.12 to 2.05)	143 (21.3)	70 (20.8)	84 (25.0)	
Q4 (≥2.05)	166 (24.7)	76 (22.6)	84 (25.0)	

**Table 2:** Association between CDAI components and risk of gastric cancer.

Variables			Univariate Logistic Regression		Multivariable Logistic Regression *	
	Cases (N = 336)	Controls (N = 336)	OR (95%CI)	*p* Value	OR (95%CI)	*p* Value
Vitamin A (μgRE)						
Q1 (<392.41)	111 (34.8)	84 (25.0)	Reference		Reference	
Q2 (392.41–505.53)	55 (16.4)	84 (25.0)	0.47 (0.30–0.73)	< 0.001	0.68 (0.42–1.10)	0.113
Q3 (505.53–673.75)	77 (22.9)	84 (25.0)	0.66 (0.43–0.99)	0.049	0.86 (0.53–1.39)	0.537
Q4 (≥673.75)	87 (25.9)	84 (25.0)	0.74 (0.49–1.12)	0.157	1.01 (0.60–1.68)	0.979
Vitamin C (mg)						
Q1 (<70.14)	125 (37.2)	84 (25.0)	Reference		Reference	
Q2 (70.14–102.38)	82 (24.4)	84 (25.0)	0.66 (0.44–0.99)	0.044	0.72 (0.47–1.12)	0.146
Q3 (102.38–142.81)	69 (20.5)	84 (25.0)	0.55 (0.36–0.84)	0.006	0.58 (0.37–0.92)	0.019
Q4 (≥142.81)	60 (17.9)	84 (25.0)	0.48 (0.31–0.74)	<0.001	0.48 (0.30–0.77)	0.002
Vitamin E (mg)						
Q1 (<6.85)	116 (34.5)	84 (25.0)	Reference		Reference	
Q2 (6.85–9.33)	70 (20.8)	84 (25.0)	0.60 (0.40–0.92)	0.019	0.89 (0.55–1.45)	0.643
Q3 (9.33–11.77)	48 (14.3)	81 (24.1)	0.43 (0.27–0.68)	<0.001	0.72 (0.42–1.24)	0.235
Q4 (≥11.77)	102 (30.4)	87 (25.9)	0.95 (0.71–1.29)	0.423	1.36 (0.76–2.43)	0.297
Zn (mg)						
Q1 (<11.83)	95 (28.3)	84 (25.0)	Reference			
Q2 (11.83–14.43)	73 (21.7)	84 (25.0)	0.77 (0.50–1.18)	0.229		
Q3 (14.43–17.40)	82 (24.4)	84 (25.0)	0.86 (0.57–1.32)	0.495		
Q4 (≥17.40)	86 (25.6)	84 (25.0)	0.91 (0.60–1.38)	0.642		
Se (μg)						
Q1 (<50.48)	123 (36.6)	84 (25.0)	Reference		Reference	
Q2 (50.48–67.37)	66 (19.6)	84 (25.0)	0.54 (0.35–0.82)	0.004	0.52 (0.32–0.83)	0.006
Q3 (67.37–84.75)	59 (17.6)	84 (25.0)	0.48 (0.31–0.74)	<0.001	0.50 (0.30–0.82)	0.006
Q4 (≥84.75)	88 (26.2)	84 (25.0)	0.72 (0.48–1.08)	0.108	0.67 (0.38–1.15)	0.147
Mn (mg)						
Q1 (<4.18)	116 (34.5)	84 (25.0)	Reference		Reference	
Q2 (4.18–5.10)	71 (21.1)	84 (25.0)	0.61 (0.40–0.93)	0.023	0.68 (0.43–1.09)	0.107
Q3 (5.10–6.64)	64 (19.0)	84 (25.0)	0.55 (0.36–0.85)	0.007	0.65 (0.39–1.08)	0.094
Q4 (≥6.64)	85 (25.4)	84 (25.0)	0.73 (0.49–1.11)	0.108	0.80 (0.49–1.32)	0.388
β-carotene (μg)						
Q1 (<3494.10)	85 (25.3)	84 (25.0)	Reference			
Q2 (3494.10–5025.91)	87 (25.9)	84 (25.0)	1.02 (0.67–1.57)	0.915		
Q3 (5025.91–8729.60)	106 (31.5)	84 (25.0)	1.24 (0.82–1.89)	0.298		
Q4 (≥8729.60)	58 (17.3)	84 (25.0)	0.68 (0.44–1.07)	0.096		

* Variables adjusted for in the multifactorial logistic regression model included vitamin A, vitamin C, vitamin E, Se, Mn, age group, marital status, smoking, and perceived daily stress level.

**Table 3:** Association between CDAI score and risk of gastric cancer.

	Model 1 *	*p* Value	Model 2 ^#^	*p* Value
CDAI				
Q1	Reference		Reference	
Q2	0.53 (0.35–0.81)	0.004	0.56 (0.36–0.87)	0.010
Q3	0.57 (0.37–0.86)	0.008	0.59 (0.38–0.90)	0.016
Q4	0.61 (0.40–0.93)	0.021	0.60 (0.39–0.92)	0.020
*p* for trend		0.020		0.019

* Model 1 was unadjusted; ^#^ Model 2 was adjusted for age group, marital status, smoking, and perceived daily stress level.

**Table 4:** Stratified analysis of the association between CDAI scores and gastric cancer risk.

Subgroups	CDAI-Q1	CDAI-Q2	*p* Value	CDAI-Q3	*p* Value	CDAI-Q4 *	*p* Value	*p*-Trend
Age groups, years								
≤55	Reference	0.65 (0.35–1.23)	0.184	0.83 (0.44–1.56)	0.562	0.60 (0.32–1.12)	0.109	0.189
>55	Reference	0.48 (0.26–0.90)	0.022	0.42 (0.23–0.76)	0.004	0.61 (0.34–1.10)	0.101	0.044
Marital status								
Married	Reference	0.67 (0.42–1.06)	0.087	0.64 (0.41–1.00)	0.052	0.65 (0.41–1.01)	0.054	0.046
Single/Separated/ Divorced/Widowed	Reference	0.08 (0.01–0.48)	0.006	0.20 (0.04–1.08)	0.061	0.33 (0.07–1.51)	0.152	0.130
Smoking								
Yes	Reference	0.60 (0.29–1.25)	0.172	0.53 (0.24–1.15)	0.106	0.50 (0.24–1.07)	0.073	0.071
No	Reference	0.54 (0.31–0.94)	0.028	0.63 (0.37–1.07)	0.087	0.68 (0.40–1.14)	0.141	0.143
Daily life stress								
None/Low	Reference	0.76 (0.43–1.36)	0.351	0.49 (0.28–0.85)	0.011	0.59 (0.35–1.00)	0.050	0.019
Moderate/High	Reference	0.37 (0.18–0.75)	0.006	0.76 (0.38–1.50)	0.427	0.65 (0.31–1.37)	0.259	0.533

* Adjusted for age group, marital status, smoking, and perceived daily stress level (excluding stratification factors).

## Data Availability

The data that support the findings of this study are available from the corresponding author, Yulan Lin, upon reasonable request. The data are not publicly available due to privacy and ethical restrictions.
